# B cell, CD8 ^+^ T cell and gamma delta T cell infiltration alters alveolar immune cell homeostasis in HIV-infected Malawian adults

**DOI:** 10.12688/wellcomeopenres.12869.3

**Published:** 2018-04-06

**Authors:** Andrew Mwale, Annemarie Hummel, Leonard Mvaya, Raphael Kamng'ona, Elizabeth Chimbayo, Joseph Phiri, Rose Malamba, Anstead Kankwatira, Henry C Mwandumba, Kondwani C Jambo

**Affiliations:** 1 Malawi-Liverpool-Wellcome Trust Clinical Research Programme, University of Malawi College of Medicine, Blantyre, Malawi; 2 Department of Clinical Sciences, Liverpool School of Tropical Medicine, Liverpool, UK

**Keywords:** BAL, HIV, B cell, CD8 T cell, gamma-delta T cells, CD4 T cell, alveolitis, adult

## Abstract

**Background**: HIV infection is associated with increased risk to lower respiratory tract infections (LRTI). However, the impact of HIV infection on immune cell populations in the lung is not well defined. We sought to comprehensively characterise the impact of HIV infection on immune cell populations in the lung.

**Methods**: Twenty HIV-uninfected controls and 17 HIV-1 infected ART-naïve adults were recruited from Queen Elizabeth Central Hospital, Malawi. Immunophenotyping of lymphocyte and myeloid cell populations was done on bronchoalveolar lavage fluid and peripheral blood cells.

**Results**: We found that the numbers of CD8 ^+^ T cells, B cells and gamma delta T cells were higher in BAL fluid of HIV-infected adults compared to HIV-uninfected controls (all p<0.05). In contrast, there was no difference in the numbers of alveolar CD4 ^+^ T cells in HIV-infected adults compared to HIV-uninfected controls (p=0.7065). Intermediate monocytes were the predominant monocyte subset in BAL fluid (HIV-, 63%; HIV+ 81%), while the numbers of classical monocytes was lower in HIV-infected individuals compared to HIV-uninfected adults (1 × 10 ^5^ vs. 2.8 × 10 ^5^ cells/100ml of BAL fluid, p=0.0001). The proportions of alveolar macrophages and myeloid dendritic cells was lower in HIV-infected adults compared to HIV-uninfected controls (all p<0.05).

**Conclusions**: Chronic HIV infection is associated with broad alteration of immune cell populations in the lung, but does not lead to massive depletion of alveolar CD4 ^+^ T cells. Disruption of alveolar immune cell homeostasis likely explains in part the susceptibility for LRTIs in HIV-infected adults.

## Introduction

HIV-infected individuals have increased susceptibility to lower respiratory tract infections (LRTIs) ^1, 2^, which account for 75–98% of lung complications in antiretroviral therapy (ART)-naïve HIV-infected adults worldwide ^3, 4^. Predisposition to LRTIs is largely attributed to HIV-induced impairment of lung immunity, including reduced frequency of respiratory antigen-specific alveolar CD4 ^+^ T cells ^5– 7^ as well as impaired alveolar macrophage function ^5, 8^. HIV infection is also associated with CD8 ^+^ T cell alveolitis, a condition characterized by the influx of HIV-specific CD8 ^+^ T cells into the lung ^9, 10^. While these immune cell perturbations partly underlie propensity for LRTIs in HIV-infected individuals, the impact of HIV infection on the composition and functions of other immune cell populations in the lung is not well defined.

Several studies have reported alterations in the proportions and functions of different immune cell populations in peripheral blood in HIV-infected individuals ^11– 14^. While peripheral blood CD4 ^+^ T cell depletion and an increase in CD8 ^+^ T cells are hallmarks of progressive untreated chronic HIV infection ^15^, depletion of B cells ^11^ and aberrant NK cell function and redistribution from CD56 ^dim^ towards CD56 ^neg^ subsets has been observed during early and chronic HIV infection ^12^. Two major human γδ T cells subsets (designated Vδ1 or Vδ2) are also altered in HIV-infected individuals, with an increase in the Vδ1 subset and a decrease in the Vδ2 subset ^13^. Furthermore, increased proportions of non-classical and intermediate monocytes and depleted myeloid and plasmacytoid dendritic cell subsets have been reported in individuals with high plasma HIV viral load ^14, 16, 17^.

We, therefore, undertook a comprehensive characterisation of the impact of HIV infection on immune cell populations in the lung. We obtained paired bronchoalveolar lavage (BAL) fluid and peripheral blood from HIV-uninfected and asymptomatic HIV-infected, antiretroviral therapy (ART)-naïve Malawian adults. We analysed and compared the proportions and numbers of CD4 ^+^ and CD8 ^+^ T cells, B cells, NK cell subsets, γδ T cells, monocytes, dendritic cell subsets, neutrophils and alveolar macrophages in samples from HIV-infected and uninfected individuals.

## Methods

### Study participants

The study was conducted at the Queen Elizabeth Central Hospital, a large teaching hospital in Blantyre, Malawi. Participants were recruited from the hospital’s voluntary counselling and testing (VCT) and ART clinics. They were adults aged ≥18yrs comprising healthy HIV-1-uninfected and asymptomatic HIV-1-infected volunteers with no clinical evidence of active disease and willing to undergo bronchoscopy and BAL for research purposes ^18^. Clinical diagnosis and radiographic examination were used to exclude individuals with active disease, including TB, from the study. No latent TB tests were carried out on the participants. The study participants were recruited from the same catchment area, with relatively similar environmental exposures. HIV testing was performed on whole blood using two commercial point-of-care rapid HIV test kits, Determine HIV 1/2 kit (Abbott Diagnostic Division) and Unigold HIV 1/2 kit (Trinity Biotech Inc.). A participant was considered HIV-uninfected if the test was negative by both kits or HIV-infected if the test was positive by both kits. If Determine and Unigold results were discordant, a third rapid test using Bioline HIV 1/2 kit (Standard Diagnostics Inc.) was performed to resolve the discordance. No baseline viral loads were done on the HIV-infected individuals. However, none of the participants were on ART at the time of recruitment to the study, but all initiated ART after sample collection according to the ‘test and treat’ Malawi national treatment guidelines. Exclusion criteria for the study were: current or history of smoking, use of immunosuppressive drugs, severe anaemia (Hb<8g/dl) and known or suspected pregnancy. The research ethics committee of Malawi College of Medicine approved the study under approval number P.03/16/1907 and all participants provided written informed consent.

### Sample collection and experimental procedures

Bronchoscopy and BAL were performed on all participants as previously described ^5, 6, 8^. Briefly, we used Instillagel to lubricate the nostrils and 2% lignocaine for vocal cords and the airways. A fiber-optic bronchoscope (Olympus, UK) was passed to the level of a sub-segmental bronchus of the right middle lobe and four 50 ml aliquots of sterile normal saline at 37°C instilled and removed using gentle hand suction. A typical BAL return from a 200ml instill is 100–140ml. The aspirated bronchoalveolar lavage fluid was placed into 50ml falcon tubes and transferred immediately to the laboratory for processing within 30 min. The fluid was filtered using sterile gauze and centrifuged at 500 x g for 10min. The supernatant was removed, the cell pellet was resuspended and washed with PBS by spinning in a centrifuge at 500 x g for 10min. The supernatant was removed and discarded while the cell pellet was resuspended in complete media. Upon microscopic examination, the cell pellet contained <5% bronchial epithelial cells or squamous cells. Due to time taken from BAL isolation and sample acquisition, there is a potential for changes in cell viability, especially neutrophils, however, the a standardised study protocol was applied to both study groups to minimise the time from isolation to acquisition. Peripheral blood was also obtained from study participants for full blood count (FBC) and peripheral blood mononuclear cell (PBMC) isolation using density gradient centrifugation. Cell counts in BAL cells and PBMCs isolated from each sample were performed using a haemocytometer.

### Immunophenotyping

Whole BAL cells (1 × 10 ^6^ cells) and PBMCs (1 × 10 ^6^ cells) were stained with predetermined optimal concentration of fluorochrome-conjugated monoclonal antibodies against human cell surface proteins. Two separate antibody panels targeting lymphocytic and myeloid cells were used. The lymphocyte panel consisted of anti-CD3 PE/Cy5, anti-CD4 Bv421, anti-CD8 APC-Cy7, anti-CD19 PE, anti-CD56 APC, anti-TCR γδ FITC, and anti-CD45 PE-CF594. The myeloid panel consisted of anti-CD45 PE-CF594, anti CD14 Bv421, anti-CD16 PE/Cy7 PC7, anti-HLADR PE/Cy5, anti-CD66 FITC, anti-CD206 APC, anti-CD11c APC/Cy7 and anti-CD123 Bv510. Further details of the antibodies are in Supplementary Table 1. All samples were analysed using a BD LSRFortessa flow cytometer (Becton Dickinson, USA).

### Statistical analysis

Statistical analyses and graphical presentation were performed using GraphPad Prism 5 (GraphPad Software, USA). We used FlowJo v10 software (Treestar, USA) to analyse flow cytometry data. The numbers of cell subsets in BAL fluid were estimated by calculating the proportion of a particular subset relative to the total number (1 × 10 ^6^ cells) of stained cells. The cell count was first calculated as number of cells relative to the BAL volume return of each individual, and this was then standardised to cells per 100ml BAL fluid as previously published ^5, 6^. In PBMCs, the absolute numbers were obtained by calculating the proportion of a particular subset relative to the full blood count (FBC) data, specifically, lymphocyte and monocytes counts. Data were analysed using Mann Whitney U test. Results are given as median and interquartile range (IQR). Differences were considered statistically significant when p<0.05.

## Results

### Study participants and samples

We recruited 20 HIV-uninfected healthy controls (median age [range] (32[18-52]; male:female, 12:8) and 17 asymptomatic HIV-infected adults (median age [range] (33 [24-58]; male:female, 8:9). The CD4 count (median [range]) was lower in HIV-infected adults compared to the HIV-uninfected controls (365[218-541]) vs. 731[541-888] cells/ul, p=0.0024). The main characteristics of the participants are summarised in Table 1. Not all experimental assays were performed on all study participants.

**Table 1.** Demographics of the study participants.

	HIV-uninfected controls (n=20)	HIV-infected ART-naive (n=17)
**Age (years), median (range)**	32(18-52)	33(24-58)
**Sex (M:F)**	12:8	8:9
**CD4 count (cells/μl), median** **(IQR)**	731(541-888)	365(218-541)

### CD8 ^+^ T cells, B cells and γδ T cells contribute to HIV-associated lymphocyte infiltration in the alveolar space

**Figure 1.**
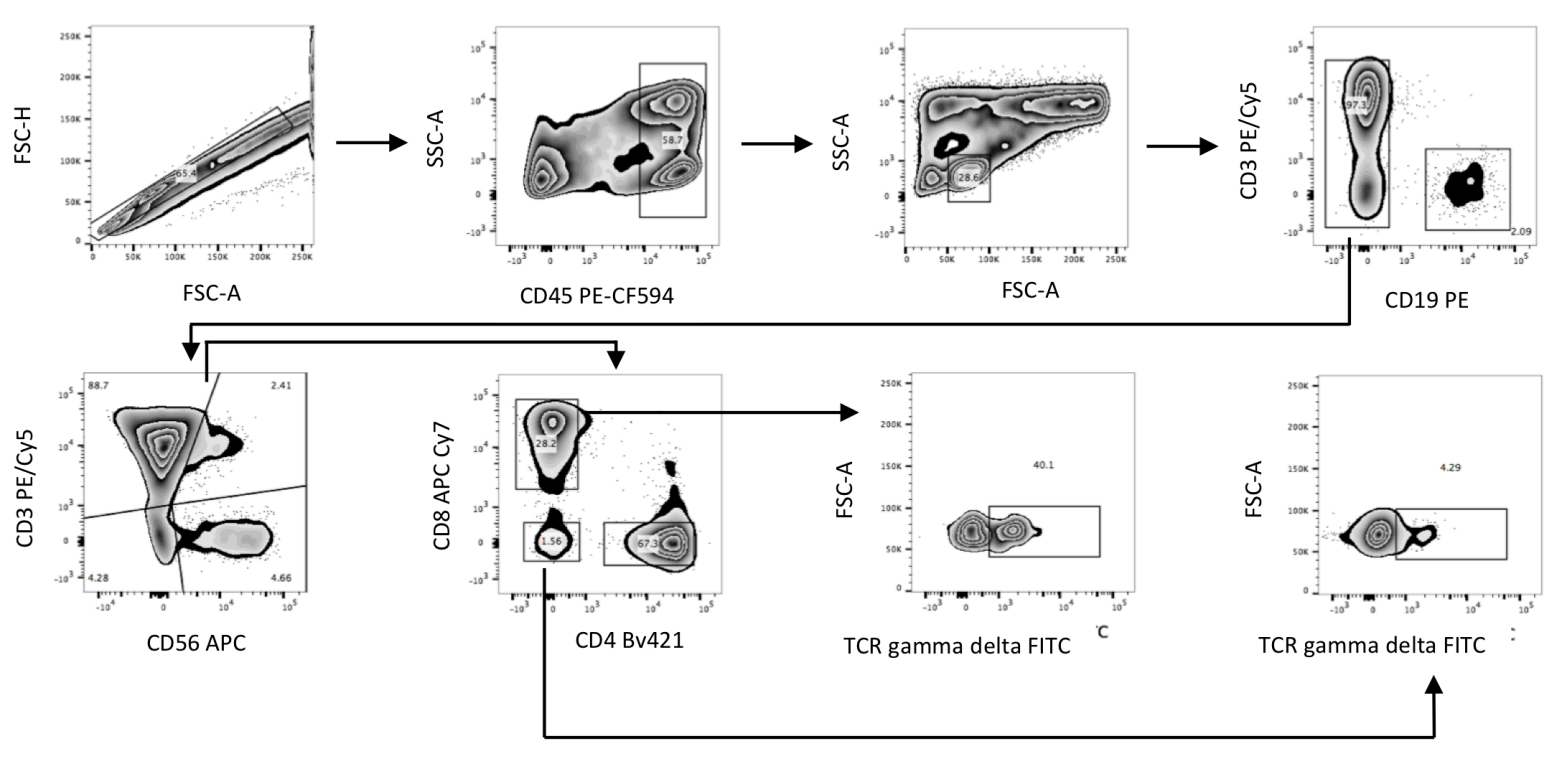
Representative flow cytometry plots for characterising lymphocytes in BAL fluid from HIV-uninfected adult. BAL cells were stained with fluorochrome-conjugated antibodies.

**Figure 2.**
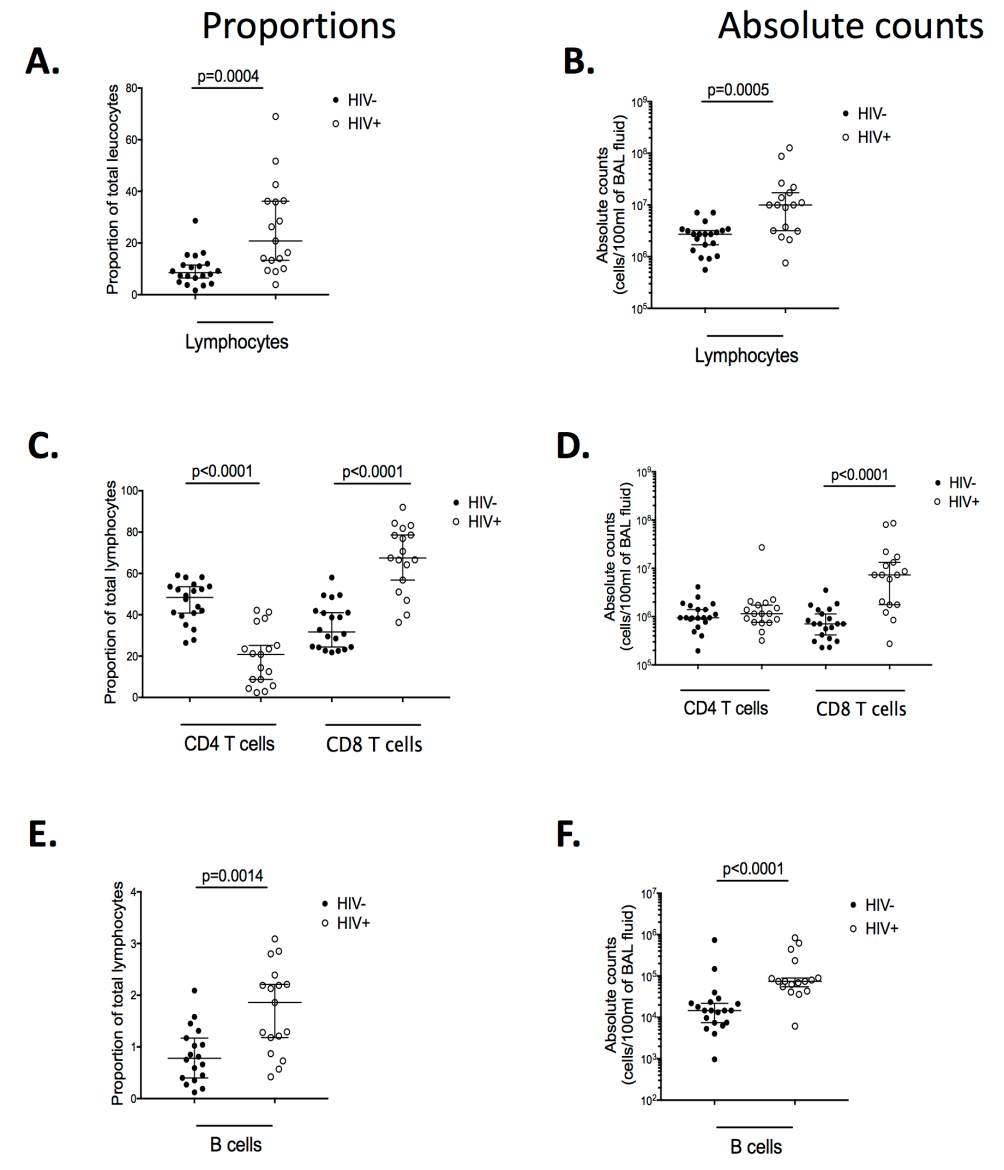
Proportions and numbers of CD4 ^+^ T cells, CD8 ^+^ T cells and CD19 ^+^ B cells in BAL fluid from ART-naïve HIV-infected compared to HIV-uninfected individuals. BAL cells were stained with fluorochrome-conjugated antibodies. **A**) Proportion of lymphocytes in BAL fluid. **B**) Numbers of lymphocytes in BAL fluid. **C**) Proportion of CD4 ^+^ and CD8 ^+^ T cells in BAL fluid. **D**) Numbers of CD4 ^+^ and CD8 ^+^ T cells in BAL fluid. **E**) Proportion of B cells in BAL fluid. **F**) Numbers of CD19 ^+^ B cells in BAL fluid. The horizontal bars represent median and 95% confidence intervals. Data were analyzed using Mann Whitney U test. (HIV-, n=20; HIV+ ART-, n=17).

**Figure 3.**
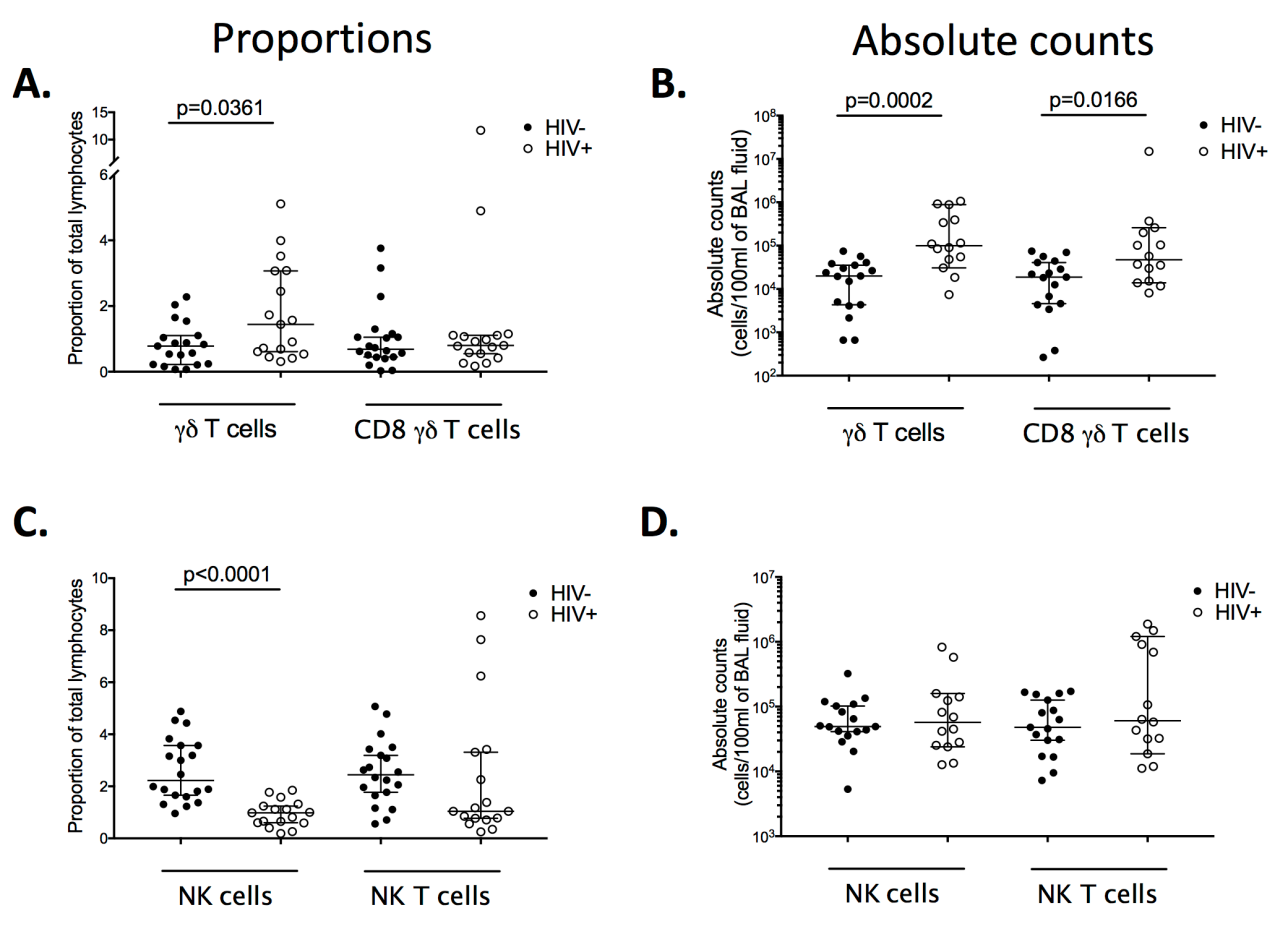
Proportions and numbers of γδ T cells and NK cells in BAL fluid from ART-naïve HIV-infected compared to HIV-uninfected individuals. BAL cells were stained with fluorochrome-conjugated antibodies. **A**) Proportion of γδ T cell subsets in BAL fluid. **B**) Numbers of γδ T cell subsets in BAL fluid. **C**) Proportion of NK cell subsets in BAL fluid. **D**) Numbers of NK cell subsets in BAL fluid. The horizontal bars represent median and 95% confidence intervals. Data were analyzed using Mann Whitney U test. (HIV-, n=20; HIV+ ART-, n=17).

We investigated the impact of HIV infection on the proportion and numbers of lymphocyte populations using flow cytometry. The gating strategy is illustrated in Figure 1. We found that the proportions and numbers of lymphocytes in BAL fluid were higher in HIV-infected adults compared to HIV-uninfected (median 20.8% vs. 8.5%, p=0.0004 and median 1 × 10 ^7^ vs. 2.7 × 10 ^6^ cells/100ml of BAL fluid, p=0.0005, respectively) ( Figure 2A and 2B). We next determined the cell subsets that were responsible for the increased frequency of lymphocytes in the alveoli. We found that the proportions and numbers of CD8 ^+^ T cells (median, 68% vs. 32%, p<0.0001 and median 7 × 10 ^6^ vs. 7 × 10 ^5^/100ml of BAL fluid, p<0.0001, respectively) and B cells (median 1.8% vs. 0.8%, p=0.0014 and median 7 × 10 ^4^ vs. 1 × 10 ^4^/100ml of BAL fluid, p<0.0001, respectively) in BAL fluid were higher in HIV-infected adults compared to HIV-uninfected controls ( Figure 2C–2F). The proportion and numbers of γδ T cells were also higher in BAL fluid from HIV-infected adults compared HIV-uninfected controls (median 1.4% vs. 0.8%, p=0.036 and median 1 × 10 ^5^ vs. 2 × 10 ^4^/100ml of BAL fluid, p=0.0002, respectively) ( Figure 3A and 3B).

In contrast, the proportions of CD4 ^+^ T cells and NK cells in BAL fluid were lower in HIV-infected adults compared to HIV-uninfected controls (CD4 ^+^ T cell, median 2% vs. 4%, p<0.0001; NK cells, median 1% vs. 2%, p<0.0001) ( Figure 2C and Figure 3C). However, the numbers showed no difference in CD4 ^+^ T cells (median 1.1 × 10 ^6^ vs. 1.0 × 10 ^6^/100 ml of BAL fluid, p=0.7065) and NK cells (median 5.4 × 10 ^4^ vs. 4.9 × 10 ^4^/100ml of BAL fluid, p=0.8911) between HIV-infected adults and HIV-uninfected controls ( Figure 2D and Figure 3D). Furthermore, there is no statistically significant difference between the alveolar CD4 T cell count in individuals with peripheral blood CD4 count of less than 350 compared to those with a peripheral blood CD4 count of greater than 350 (Median [Interquartile range] 1.9 × 10 ^6^ [0.9-2.2 × 10 ^6^] vs. 0.9 × 10 ^6^[0.4-2.2 × 10 ^6^]/100ml of BAL fluid, p=0.1905). These findings demonstrate that HIV infection has a differential impact on alveolar lymphocyte populations.

### Differential impact of HIV infection on lymphocyte subsets in the alveolar and blood compartments

We then investigated the similarities and differences of HIV-associated changes in cell composition between BAL fluid and peripheral blood. In agreement with BAL fluid, the proportions of CD8 ^+^ T cells in peripheral blood were higher in HIV infected adults compared to HIV-uninfected controls (Median 47% vs. 24%, p<0.0001) ( Supplementary Figure 1). The proportions of CD4 ^+^ T cells in peripheral blood were lower in HIV-infected adults compared to HIV-uninfected controls (Median 20% vs. 46%, p<0.0001) ( Supplementary Figure 1). In contrast with BAL fluid, the proportion of B cells in peripheral blood was lower in HIV-infected adults compared to HIV-uninfected controls (Median 5.8% vs. 9.4%, p=0.0472) ( Supplementary Figure 1). The proportion of CD3 ^+^CD56 ^+^ NK T cells in peripheral blood was lower in HIV-infected adults compared to HIV-uninfected controls (Median 0.03% vs. 0.09%, p=0.0386) ( Supplementary Figure 1). The proportion of CD8 γδ T cells in peripheral blood was higher in HIV-infected adults compared to HIV-uninfected controls (Median 1.9% vs. 0.76%, p=0.0229) ( Supplementary Figure 1). The findings show that HIV infection differentially impacts lymphocyte populations in the alveolar space and peripheral blood compartments.

### Differential impact of HIV infection on monocyte subsets in the alveolar and blood compartments

**Figure 4.**
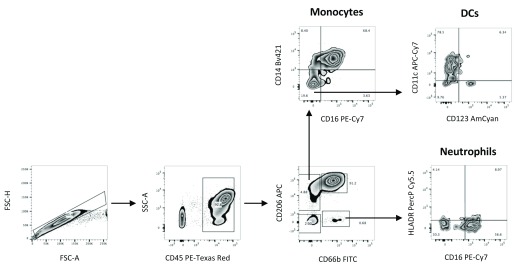
Representative flow cytometry plots for characterising myeloid cells in BAL fluid from an HIV-uninfected adult. BAL cells were stained with fluorochrome-conjugated antibodies.

**Figure 5.**
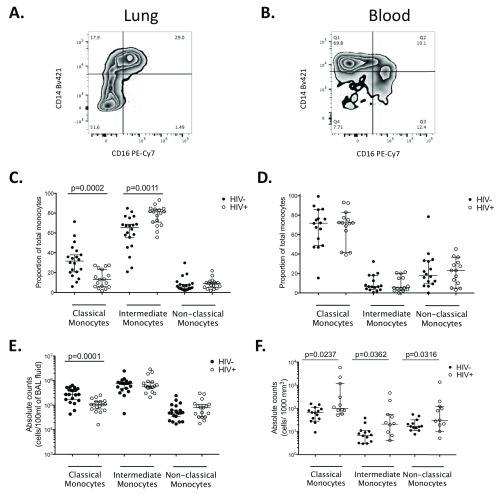
Proportions and numbers of monocyte subsets in BAL fluid and peripheral blood from ART-naïve HIV-infected compared to HIV-uninfected individuals. BAL cells and PBMCs were stained with fluorochrome-conjugated antibodies. **A**) Flow cytometry representative plot of stained BAL sample from an HIV-uninfected control. **B**) Flow cytometry representative plot of stained peripheral blood sample from an HIV-uninfected control. **C**) Proportion of monocytes subsets in BAL fluid. **D**) Proportion of monocyte subsets in peripheral blood. **E**) Numbers of monocytes subsets in BAL fluid. **F**) Numbers of monocyte subsets in peripheral blood.The horizontal bars represent median and 95% confidence intervals. Data were analyzed using Mann Whitney U test. (BAL fluid, HIV-, n = 20; HIV+ ART-, n = 17; PBMC, HIV-, n=16; HIV+ ART-, n=14).

Next, we investigated the impact of HIV infection on the monocyte population in BAL fluid compared to peripheral blood. The gating strategy is illustrated in Figure 4. First we determined the composition of the monocyte cell population in BAL fluid in comparison to peripheral blood. We found that irrespective of HIV status CD14 ^+^ CD16 ^+^ intermediate monocytes were the predominant subset in BAL fluid, followed by CD14 ^++^CD16 ^lo^ classical monocytes and then CD14 ^lo^CD16 ^+^ non-classical monocytes (HIV-, Median 63% vs. 33% vs. 5%; HIV+, Median 81% vs. 13% vs. 9%) ( Figure 5A and 5C). In blood, irrespective of HIV status, CD14 ^++^ CD16 ^lo^ classical monocytes were the predominant monocyte subset, followed by CD14 ^lo^CD16 ^+^ non-classical monocytes and then CD14 ^+^CD16 ^+^ intermediate monocytes (HIV-, median 74% vs. 18% vs. 9%; HIV+, median 73% vs. 23% vs. 8%) ( Figure 5B and 5D).

Second, we compared the proportions and numbers of monocyte population in BAL fluid and peripheral blood between HIV-infected adults and HIV-uninfected controls. In BAL fluid, we found that the proportion and numbers of CD14 ^+^CD16 ^lo^ classical monocytes were lower in HIV-infected adults compared to HIV-uninfected controls (median 13% vs. 33%, p=0.0002 and median 1 × 10 ^5^ vs. 2.8 × 10 ^5^ cells/100ml of BAL fluid, p=0.0001, respectively) ( Figure 5C and 5E). In contrast, the proportion of CD14 ^+^CD16 ^+^ intermediate monocytes was higher in HIV-infected adults compared the HIV-uninfected controls (median, 80% vs. 64%, p=0.0011) but the numbers were similar between the two groups (median 6.0 × 10 ^5^ vs. 7.7 × 10 ^5^ cells/100ml of BAL fluid, p=0.8628) ( Figure 5C and 5E). In blood, we found that the numbers of CD14 ^+^ CD16 ^lo^ classical monocytes (median 110 vs. 60 cells/1000 mm ^3^, p=0.0237), CD14 ^+^CD16 ^+^ intermediate monocyets (median 20 vs. 6 cells/1000 mm ^3^, p=0.0362) and CD14 ^lo^CD16 ^+^ non classical monocytes (median 30 vs. 10 cells/1000 mm ^3^, p=0.0316) were higher in HIV-infected adults compared to HIV-uninfected controls ( Figure 5F). These findings underscore differences in the composition and the impact of HIV infection on immune cells in the lung and systemic compartments.

### Altered proportions of alveolar macrophages and dendritic cell populations in HIV-infected adults

**Figure 6.**
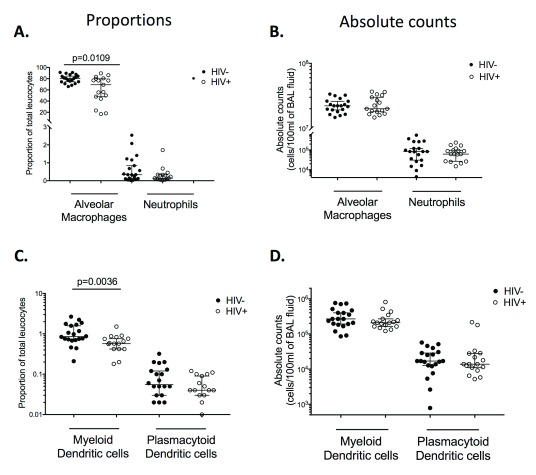
Proportions and numbers of alveolar macrophages, neutrophils and dendritic cells in BAL fluid from ART-naïve HIV-infected compared to HIV-uninfected individuals. BAL cells were stained with fluorochrome-conjugated antibodies. **A**) Proportion of alveoalar macrophages and neutrophils in BAL fluid. **B**) Numbers of alveoalar macrophages and neutrophils in BAL fluid. **C**) Proportion of dendritic cell subsets in BAL fluid. **D**) Numbers of dendritic cell subsets in BAL fluid. The horizontal bars represent median and 95% confidence intervals. Data were analyzed using Mann Whitney U test. (HIV-, n = 20; HIV+ ART-, n = 17).

Lastly, we investigated the impact of HIV on alveolar macrophages (AM), neutrophils and dendritic cell populations in BAL fluid. We found that the proportions of alveolar macrophages and myeloid dendritic cells were lower in HIV infected adults compared to HIV-uninfected controls (AM, median 73% vs. 80%, p=0.0109; mDC, median 0.6% vs. 0.9%, p=0.0036) ( Figure 6A and 6C). The proportion of neutrophils and plasmacytoid dendritic cells was similar between HIV-infected adults and HIV-uninfected controls (neutrophils, median 0.34% vs. 0.14%, p=0.0789; pDC, median 0.04% vs. 0.05%, p=0.1947) ( Figure 6A and 6C). The numbers of alveolar macrophages (median 2.0 × 10 ^6^ vs. 2.2 × 10 ^6^ cells/100ml of BAL fluid, p=0.8628), neutrophils (median 6.2 × 10 ^4^ vs. 8.4 × 10 ^4^ cells/100ml of BAL) and dendritic cells (mDC, median 2.1 × 10 ^5^ vs. 2.7 × 10 ^5^ cells/100ml of BAL fluid, p=0.2676; pDC, median 1.3 × 10 ^5^ vs. 1.7 × 10 ^5^ cells/100ml of BAL fluid, p=0.5328) were similar between the HIV-infected adults and HIV-uninfected controls ( Figure 6B and 6D). Taken together, the findings show that chronic HIV-infection is associated with a disruption in the homeostatic proportions of alveolar macrophage and dendritic cell populations.

## Discussion

We report the broad impact of HIV infection on immune cell populations in the alveolar space beyond the well-characterised CD8 ^+^T cell alveolitis observed in previous studies ^5, 6, 9, 10^. We show that in addition to CD8 ^+^ T cells, B cells and γδ T cells are increased, while classical monocytes are decreased in BAL fluid from ART-naïve HIV-infected adults compared to HIV-uninfected individuals. We further show generalised disruption in the proportions of immune cell subsets including alveolar macrophages, CD4 ^+^ T cells, myeloid dendritic cells, intermediate monocytes and NK cells in BAL fluid of asymptomatic chronic HIV-infected adults.

Although HIV-infection was associated with accumulation of B cells and γδ T cells in BAL fluid, their contribution to pulmonary immunity during chronic HIV infection is incompletely understood. However, previous studies have reported HIV-associated impairment of function of these two cell subsets in peripheral blood ^19– 21^. Consistent with what has been observed in the systemic circulation, hyperglobulinemia has been reported in BAL fluid of HIV-infected adults ^22, 23^, but the antibodies have impaired opsonic function ^24^. It is plausible that the HIV-associated increase in B cells in the lung results in increased antibody production and BAL fluid hyperglobulinemia. Furthermore, the increase in γδ T cells that we found in the present study supports the findings of Agostini *et. al.* ^25^, who showed that HIV-infected individuals with CD8 ^+^ T cell alveolitis had increased γδ T cells in BAL fluid, which were predominantly of the Vδ2 subset. However, HIV infection was also associated with anergic γδ T cells that were characterised by their substantially deficient response to phosphoantigens ^26– 28^. Taken together, the findings of previous studies lead us to postulate that despite the increase in numbers, lung B cells and γδ T cells from HIV-infected individuals have impaired function as their blood counterparts.

HIV infection is associated with massive depletion of mucosal CD4 ^+^ T cells in the gut ^29, 30^ and gradual decline in peripheral blood CD4 ^+^ T cells ^15^. We have shown preserved mucosal CD4 ^+^ T cells in BAL fluid from chronic HIV-infected adults, even in those with depleted peripheral blood CD4 ^+^ T cells. Our findings are consistent with previous work that showed lung CCR5 ^+^CD4 ^+^ T cells are not massively depleted during HIV infection ^31^. However, it is not clearl whether these preserved mucosal CD4 ^+^ T cells in BAL fluid would also be true in individuals with symptomatic chronic HIV infection. The mechanisms behind this preservation of alveolar CD4 ^+^ T cells is unclear and warrants further investigation. However, Mahlknecht *et. al.* has shown that macrophages can prevent CD4 ^+^ T cell apoptosis *in vitro* via cell to cell contact using a mechanism that involves stimulation of *nef*-expressing CD4+ T cells with macrophage membrane-bound TNF ^32^. Nef in presence of TNF stimulation promotes activation of anti-apoptotic transcription factor NF-κB, resulting in blockade of caspase-8 activation and subsequent apoptosis ^32^. It is therefore plausible that alveoalar macrophages could promote survival of CD4 ^+^ T cells in the lung through similar mechanisms, but this warrants further investigation. However, although alveolar CD4 ^+^ T cells are not massively depleted during chronic HIV infection, their functional capacity is perturbed ^5– 7^.

Consistent with others ^33– 35^, we have showed that CD16 ^+^CD14 ^+^ intermediate monocytes were the predominant subset in BAL fluid. The lower proportion of CD14 ^+^CD16 ^lo^ classical monocytes in BAL compared to peripheral blood is consistent with work from Baharom *et al.*, which comprehensively characterized monocyte subsets and their function in the lung mucosa ^36^. CD16 ^+^ monocytes and AM have been shown to be permissive to HIV infection ^8, 37^. The abundance of intermediate monocytes and AM in BAL fluid increases potential cellular targets for HIV. Our findings that AM are preserved during chronic HIV infection, may partly be attributed to the long life span of these cells ^38, 39^, as well as their resistance to the cytopathic effects of HIV ^40, 41^. In contrast, we observed a depletion in classical monocytes in BAL fluid from HIV-infected individuals. The mechanism for the selective depletion of classical monocytes is unclear, but might involve HIV-induced apoptosis ^42^ or loss/downregulation of surface CD14 ^43^. In steady state, alveolar macrophages originate from erythro-myeloid progenitors (EMPs), while monocytes originate from haematopoietic stem cells (HSCs) ^44^, hence the differential impact of HIV on these subsets might be due to the distinct nature of their source of origin. On the other hand, during an inflammatory state, classical monocytes are thought to differentiate into lung macrophages and contribute to clearance of invading pathogens ^45^. Presence of a wide array of HIV-permissive cells in the lung, including recruited and resident cells, could contribute to maintenance of local viral production and subsequent disruption of immune cell populations and homeostasis in this compartment.

A potential limitation of the study is that the numbers of BAL cell subsets are extremely difficult to measure with a very high degree of accuracy due to the variations in the dilution factor of epithelial lining fluid and differences in BAL fluid volume return. However, using a method utilised in previous studies ^5, 6^, we calculated numbers of cell subsets using the BAL cell count obtained from a haemocytometer combined with proportions obtained by immunophenotyping. We have confidence in the reliability of this method to measure the numbers for the other cell subsets, as we have replicated the observation that the absolute number of CD8 ^+^ T cells is higher in HIV-infected adults compared with HIV-uninfected individuals ^5, 6, 9, 10^. Furthermore, using data from our previous work that focused on measuring cytokines in BAL fluid, we found no statistically significant difference in concentration of urea in BAL fluid between HIV-infected adults compared to HIV-uninfected individuals, suggesting that permeability of the alveolar space might not be different in the two groups (unpublished).

In conclusion, our findings show that HIV infection is associated with broad alteration of immune cell populations in the lung. Disruption in immune homeostasis has been shown to lead to increased susceptibility to both infectious and non-infectious diseases. The broad alteration of immune cell populations in the lung in part explain the propensity to LRTI in HIV-infected individuals. However, the degree to which successful anti-retroviral therapy restores the composition of immune cells in the lung warrants further investigation.

## Data availability

The data underlying the results presented in this manuscript are available from OSF: osf.io/ykve4 ^46^.

## Supplementary material

**Supplementary Figure 1. Proportions of CD4 ^+^ T cells, CD8 ^+^ T cells, CD19 ^+^ B cells, γδ T cells and NK in peripheral blood from ART-naïve HIV-infected compared to HIV-uninfected individuals.** PBMC were stained with fluorochrome-conjugated antibodies. A) Proportion of CD4 ^+^ and CD8 ^+^ T cells in peripheral blood. B) Proportion of B cells in peripheral blood. C) Proportion of γδ T cell subsets in peripheral blood. D) Proportion of NK cell subsets in peripheral blood. The horizontal bars represent median and 95% confidence intervals. Data were analyzed using Mann Whitney U test. (HIV-, n=16; HIV+ ART-, n=14).

**Supplementary Figure 2. Representative flow cytometry plots for characterising monocytes in PBMCs.**

**Supplementary Figure 3. Representative flow cytometry plots for characterising myeloid in BAL fluid.**

**Supplementary Figure 4. Representative flow cytometry plots for characterising dendritic cell subsets in BAL fluid.**

**Supplementary Table 1. Details of fluorochrome-conjugated antibodies used in the study.**
