# Supplementary figures and images for: B cell, CD8 + T cell and gamma delta T cell infiltration alters alveolar immune cell homeostasis in HIV-infected Malawian adults
Source: Wellcome Open Res. 2018 Apr 6;2:105. Originally published 2017 Oct 27. [Version 3] doi: 10.12688/wellcomeopenres.12869.3 (PMC5872007; doi:10.12688/wellcomeopenres.12869.3)

Supplementary Figure 1.

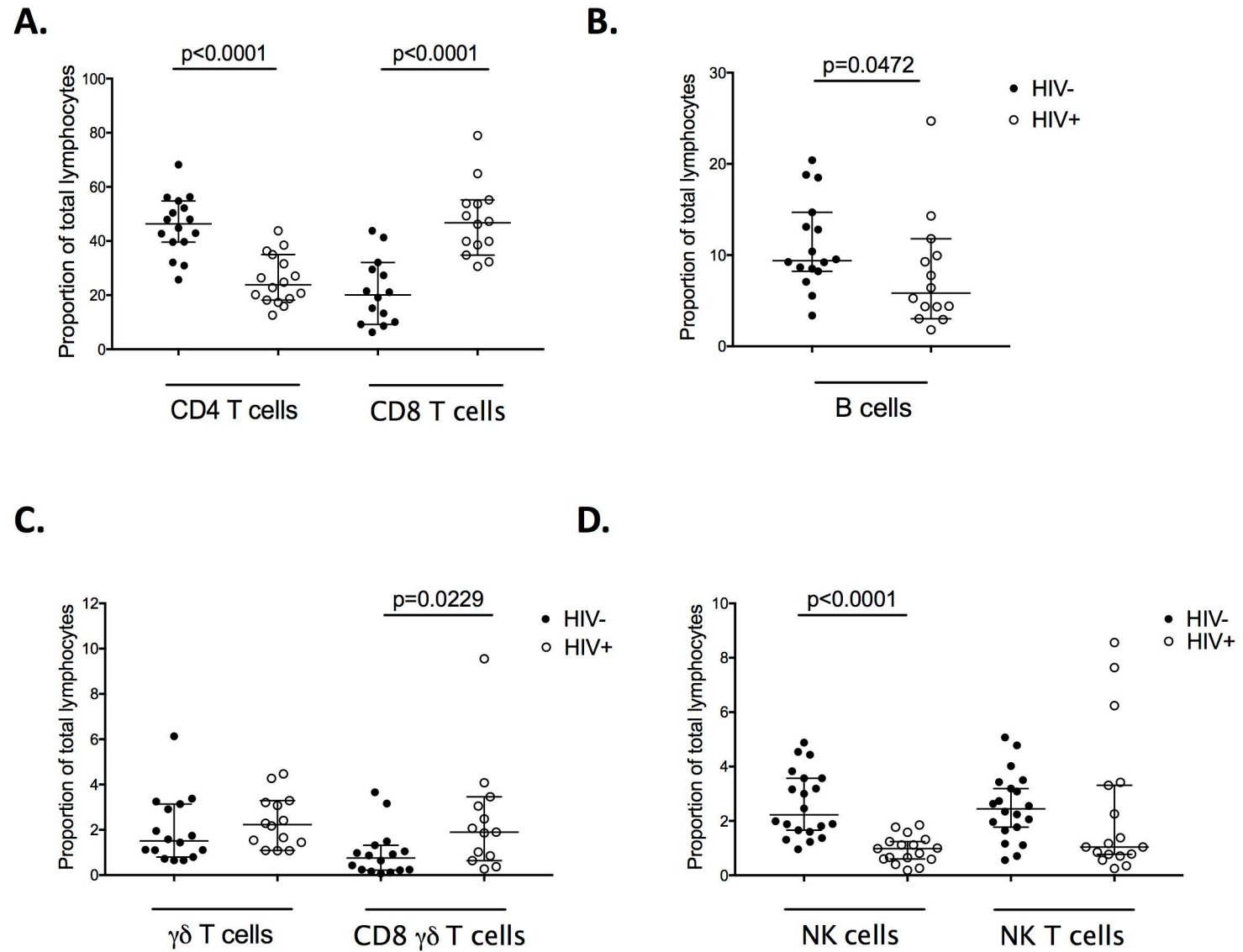

Supplement: Supplementary file 1 [file wellcomeopenres-2-15734-s0001.tgz › 33d3363b-0c18-412c-86c2-48e806515efb.pdf]

Supplementary Figure 2.

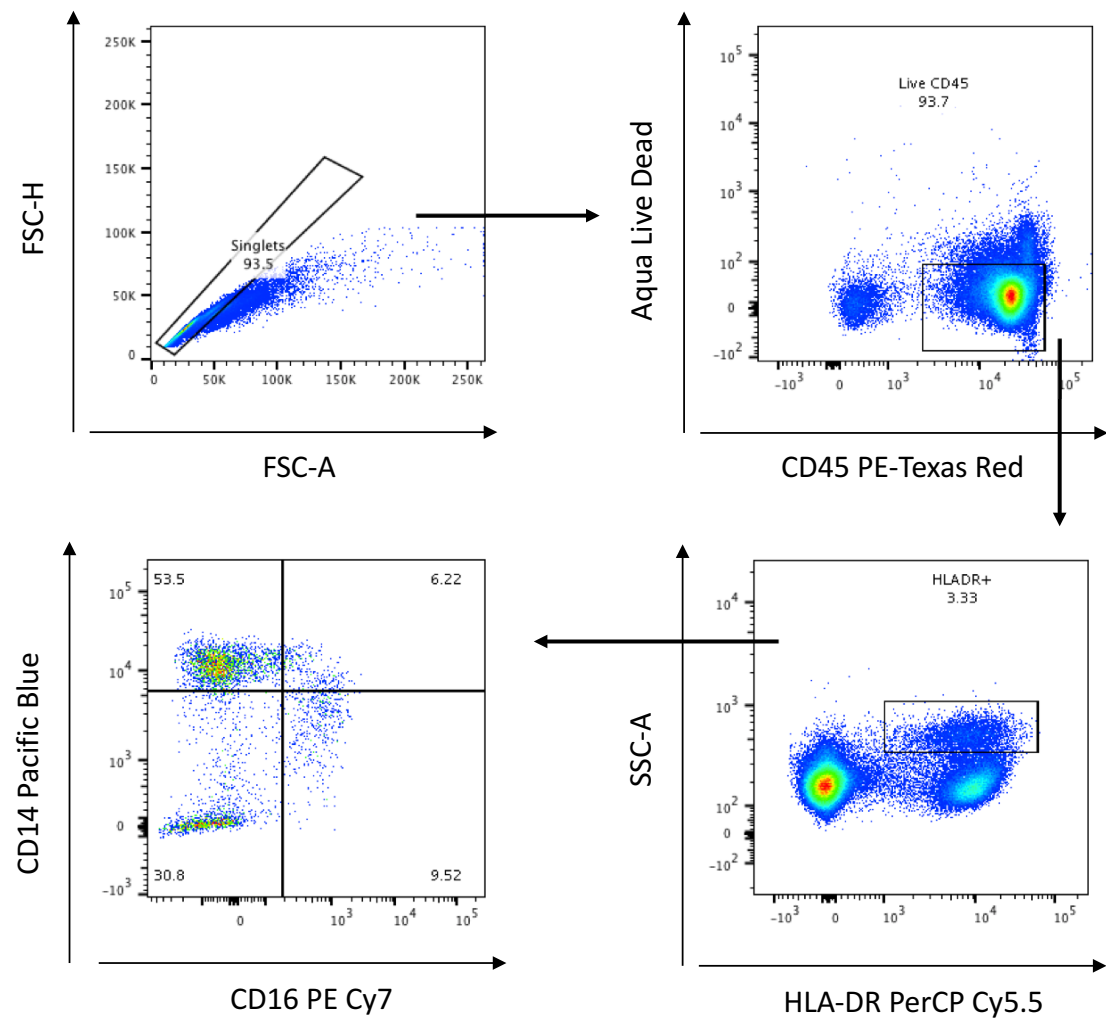

Supplement: Supplementary file 2 [file wellcomeopenres-2-15734-s0002.tgz › d2921e9e-6478-4dad-9913-d8e271d87d98.pdf]

Supplementary Figure 3.

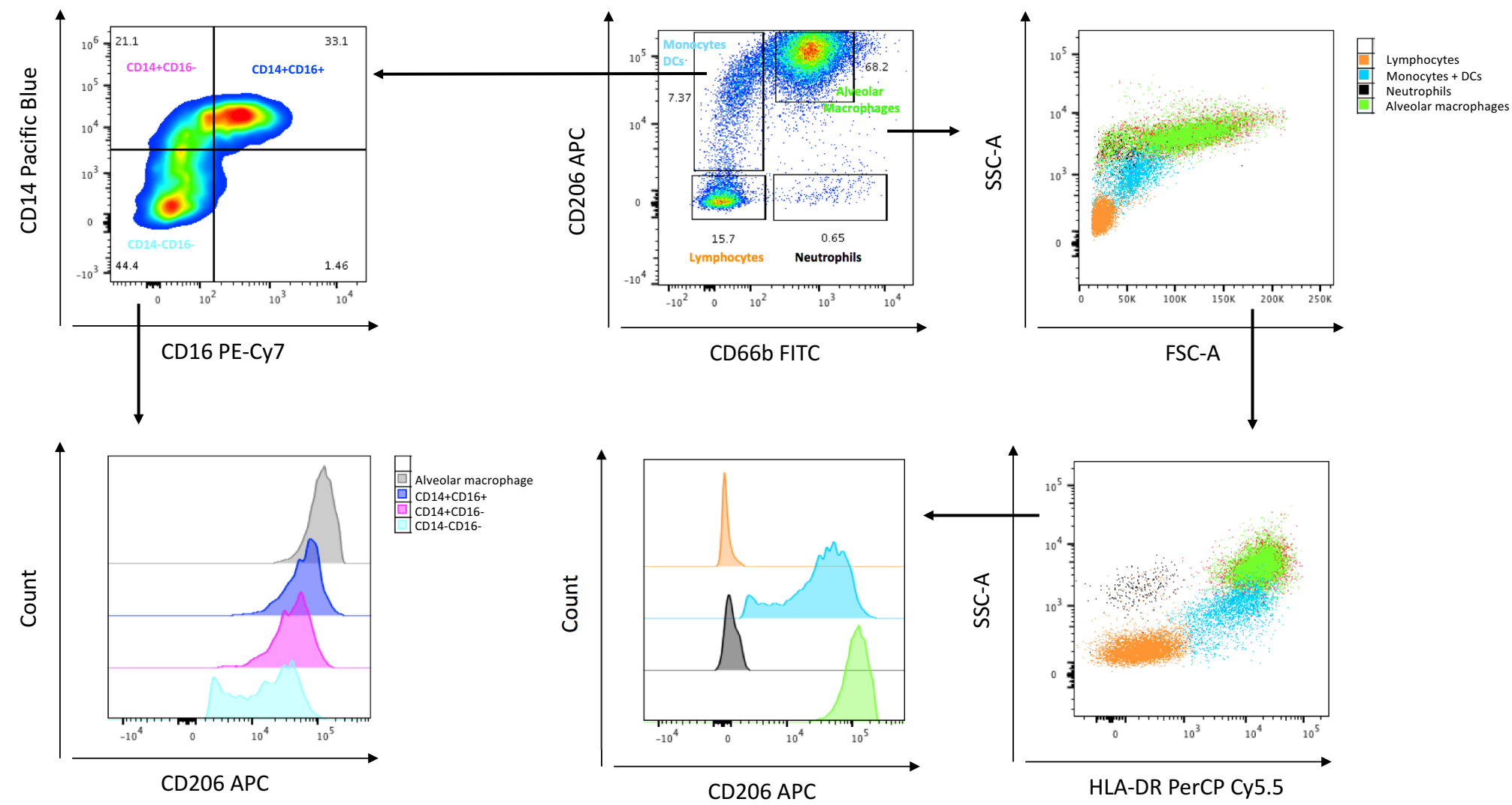

Supplement: Supplementary file 3 [file wellcomeopenres-2-15734-s0003.tgz › a183e71b-b813-4465-b897-7ab00a776be3.pdf]

Supplementary Figure 4.

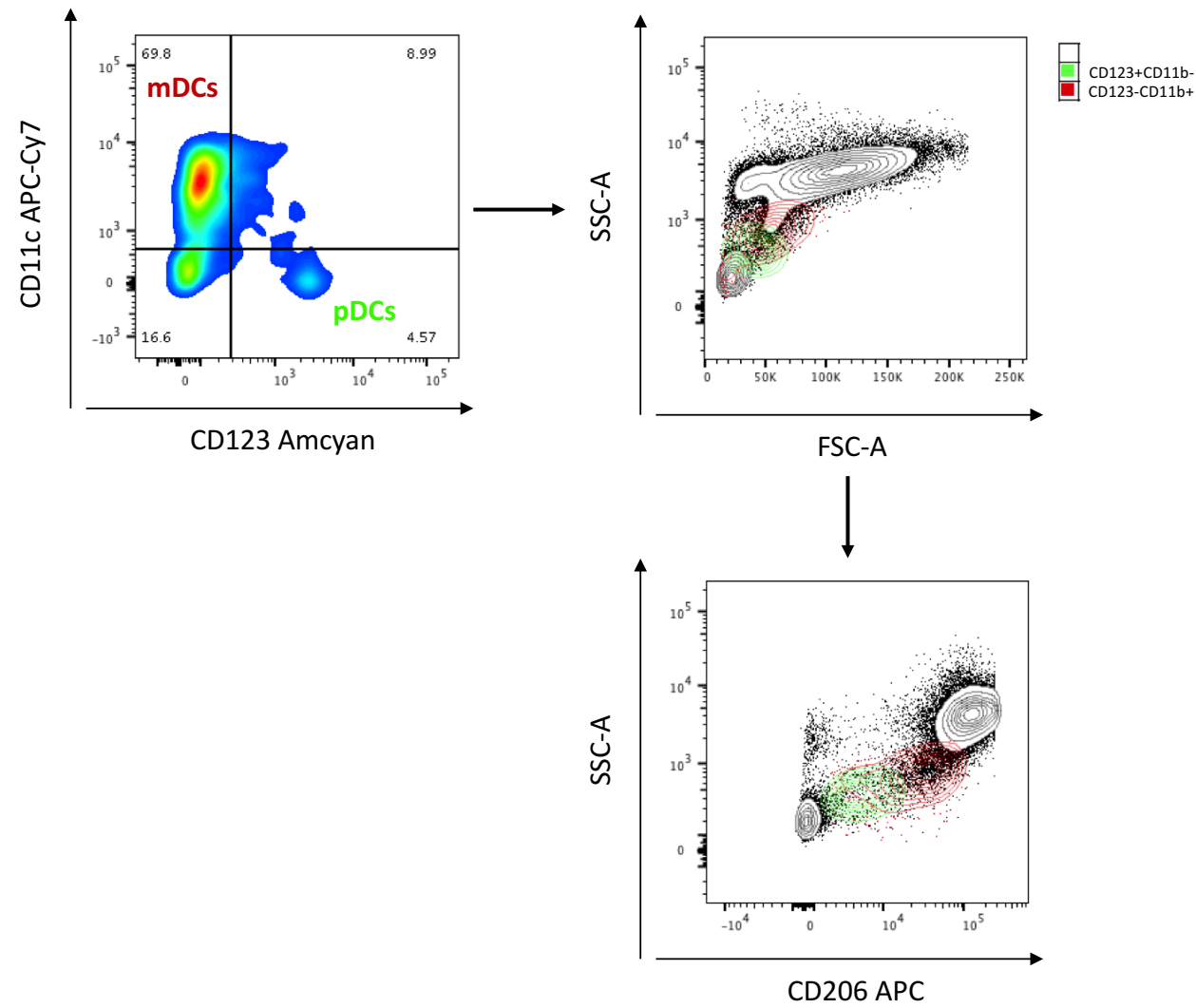

Supplement: Supplementary file 4 [file wellcomeopenres-2-15734-s0004.tgz › ba73879e-d04b-4e9a-9d80-d15e9397b53d.pdf]
