# Supplementary material for: B cell, CD8 + T cell and gamma delta T cell infiltration alters alveolar immune cell homeostasis in HIV-infected Malawian adults
Source: Wellcome Open Res. 2018 Apr 6;2:105. Originally published 2017 Oct 27. [Version 3] doi: 10.12688/wellcomeopenres.12869.3 (PMC5872007; doi:10.12688/wellcomeopenres.12869.3)
Supplement: Supplementary file 5 [file wellcomeopenres-2-15734-s0000.tgz › 070b8745-c202-48c9-8c98-a55e5b5c62c0.pdf]

Supplementary Table 1: Details of fluorochrome-conjugated antibodies used in the study.

Antibody	Species Ab was raised	Manufacturer	Cat. number	Clone
anti-CD3 PE/Cy5	Mouse IgG1, k	Biolegend	300410	UCHT1
anti-CD4 Bv421	Mouse IgG1, k	Biolegend	300532	RPA-T4
anti-CD8 APC-Cy7	Mouse IgG1, k	Biolegend	300926	HIT8a
anti-CD19 PE	Mouse IgG1, k	Biolegend	302208	HIB19
anti-CD56 APC	Mouse IgG1, k	Biolegend	318310	HCD56
anti-TCR γδ FITC	Mouse IgG1, k	Biolegend	331208	B1
anti-CD45 PE-CF594	Mouse IgG1, k	BD Biosciences	562279	HI30
anti CD14 Bv421	Mouse IgG1, k	Biolegend	325628	HCD14
anti-CD16 PE/Cy7 PC7	Mouse IgG1, k	Biolegend	302016	3G8
anti-HLADR PE/Cy5	Mouse IgG2a, k	Biolegend	307608	L243
anti-CD66 FITC	Mouse IgM, k	Biolegend	305104	G10F5
anti-CD206 APC	Mouse IgG1, k	Biolegend	321110	15-2
anti-CD11c APC/Cy7	Mouse IgG1, k	Biolegend	337218	Bu15
anti-CD123 Bv510	Mouse IgG1, k	Biolegend	306022	6H6

Volume Used (per 100ul)
5ul
2.5ul
5ul
2.5ul
5ul
5ul
5ul
2.5ul
5ul
5ul
2.5ul
5ul
5ul
5ul
