# Supplementary material for: B cell, CD8 + T cell and gamma delta T cell infiltration alters alveolar immune cell homeostasis in HIV-infected Malawian adults
Source: Wellcome Open Res. 2018 Apr 6;2:105. Originally published 2017 Oct 27. [Version 3] doi: 10.12688/wellcomeopenres.12869.3 (PMC5872007; doi:10.12688/wellcomeopenres.12869.3)
Supplement: Peer review history

# Peer review: Referee response for version 3

**DOI:** 10.21956/wellcomeopenres.15734.r32857

**Published:** 2018-04-06

**Authors:** Dawn M.E. Bowdish

**Affiliations:** 1 Department of Pathology and Molecular Medicine, McMaster University, Hamilton, ON, Canada

The authors have made all the necessary revisions and addressed all my concerns. I appreciate them pointing out the differences in monocyte composition between European and African populations as I was not aware that a comparative study had been done. I am very supportive of seeing this work published.

I have read this submission. I believe that I have an appropriate level of expertise to confirm that it is of an acceptable scientific standard.

# Peer review: Referee response for version 2

**DOI:** 10.21956/wellcomeopenres.14695.r31691

**Published:** 2018-03-27

**Authors:** Dawn M.E. Bowdish

**Affiliations:** 1 Department of Pathology and Molecular Medicine, McMaster University, Hamilton, ON, Canada

The applicants have clearly demonstrated differences in the immune compostion of the lungs in HIV+ and negative individuals. Overall, I am very supportive of this work but I have a few minor concerns.

 Methodolological concerns:

 My only concern is in the immunophenotyping of myeloid cells in blood and BAL. It would be helpful to label the gating strategy in Fig 4 with what cell types each group represents (e.g. mDC, monocyte). Is the gating strategy for the lung and blood the same? The proportions of the three monocyte subsets in blood looks slightly different than reported elsewhere and I wonder if this is due to differences in gating? Specifically intermediate monocytes are HLADR+ and it looks like in Fig 4, this is not a component of the gating strategy? CD11c is also found on circulating monocytes (esp non-classical) – is it possible that these are included in the DC gates rather than the monocyte gates? I find the gating strategy in Patel et al. Journal of Experimental Medicine Jun 2017, jem.20170355; DOI: 10.1084/jem.20170355 is consistent for monocytes in blood and tissues. It also appears that the monocytic proportion of the BAL are gated first on CD206 – although CD206 is found on alveolar and interstitial alveolar macrophages, I’m unaware of it being expressed on lung monocytes and so this might explain the discrepancy.

 Grammar: In the heading “Sample collection and experimental procedures, the speeds at which the samples are centrifuged at should be “x g” not “g” (otherwise you are measuring in grams).

I have read this submission. I believe that I have an appropriate level of expertise to confirm that it is of an acceptable scientific standard, however I have significant reservations, as outlined above.

# Response

**Published:** 2018-03-29

**Authors:** Kondwani Jambo

**Affiliations:** Malawi-Liverpool-Wellcome Trust Clinical research programme, Malawi

**Reviewer comment:** My only concern is in the immunophenotyping of myeloid cells in blood and BAL. It would be helpful to label the gating strategy in Fig 4 with what cell types each group represents (e.g. mDC, monocyte).

**Response: **We have labelled the gating strategy in Figure 4

**Reviewer comment: **Is the gating strategy for the lung and blood the same?

**Response: **No. We utilised two different gating strategies for the blood and lung, as the composition of the cells in the two compartments is different. 

**Reviewer comment: **The proportions of the three monocyte subsets in blood looks slightly different than reported elsewhere and I wonder if this is due to differences in gating?

**Response: **The population of monocytes in blood is identified using standard gating (see Supplementary Figure 2). We have data from a similar cohort (unpublished), in which we made a direct comparison of monocyte subsets in an African cohort compared to European cohort, which shows that the proportions of the monocyte subsets in blood are different in the two groups. Specifically, the proportion of classical monocytes is higher in European individuals compared to African individuals, and the proportion of non-classical monocytes is higher in the African individuals compared to European individuals.

**Reviewer comment: **Specifically intermediate monocytes are HLADR+ and it looks like in Fig 4, this is not a component of the gating strategy?

**Response: **We can confirm that the monocyte population was HLADR positive. See plots in Supplementary Figure 3

**Reviewer comment: **CD11c is also found on circulating monocytes (esp non-classical) – is it possible that these are included in the DC gates rather than the monocyte gates?

**Response: **We have noted an error in the presentation of the gating strategy. The DC gate actually is derived from the monocyte plot (CD14 ^lo/- ^CD16 ^-^ population), and not from the CD206 ^-^ CD66b ^-^ population. The error on gating strategy has now been corrected (See Figure 4). 

 Furthermore, we acknowledge that the Myeloid DCs population might not be pure. However, upon further examination of the cells denoted as dendritic cells, we found that the cells are smaller than alveolar macrophages, less granular than neutrophils and exhibit low expression for CD206 (in comparison to alveolar macrophages) (see Supplementary Figure 4). 

**Reviewer comment: **I find the gating strategy in Patel et al. Journal of Experimental Medicine Jun 2017, jem.20170355; DOI: 10.1084/jem.20170355 is consistent for monocytes in blood and tissues. It also appears that the monocytic proportion of the BAL are gated first on CD206 – although CD206 is found on alveolar and interstitial alveolar macrophages, I’m unaware of it being expressed on lung monocytes and so this might explain the discrepancy.

**Response: **Thank you for the manuscript. We agree peripheral blood monocytes do not express CD206. However, we know that when monocytes are differentiating into macrophages they gain expression of CD206. We suspect the expression of CD206 on the monocytes in BAL might be related to their differentiation process into monocyte-derived macrophages. According to Supplementary Figure 3, both subsets express CD206 at lower levels than alveolar macrophages, but CD14 ^+^CD16 ^+ ^monocytes express higher levels of CD206 compared to CD14 ^+^CD16 ^-^.

**Reviewer comment: **Grammar: In the heading “Sample collection and experimental procedures, the speeds at which the samples are centrifuged at should be “x g” not “g” (otherwise you are measuring in grams).

**Response:** We agree with the reviewer. We have corrected the typo

# Peer review: Referee response for version 2

**DOI:** 10.21956/wellcomeopenres.14695.r29252

**Published:** 2017-12-22

**Authors:** David H. Dockrell

**Affiliations:** 1 MRC Centre for Inflammation Research, University of Edinburgh, Edinburgh, UK

The revised manuscript has addressed the reviewers comments  for the most part. It is still not clear from the rather brief responses what if anyhting has been done to resolve the queries about HLA DR staining and use of CR2 staining etc. The answer may well be nothing further was possible to address this but some clarification would have been helpful unless I have missed this response. Otherwise it seems the authors have carefully addressed the comments.

I have read this submission. I believe that I have an appropriate level of expertise to confirm that it is of an acceptable scientific standard.

# Peer review: Referee response for version 1

**DOI:** 10.21956/wellcomeopenres.13949.r27371

**Published:** 2017-11-27

**Authors:** Graeme Meintjes, Muki Shey

**Affiliations:** 1 Groote Schuur Hospital, University of Cape Town, Cape Town, South Africa; 2 Department of Medicine, University of Cape Town, Cape Town, South Africa

**Review by Graeme Meintjes**

 Mwale and colleagues report a cross-sectional study conducted in Blantyre, Malawi, in which 17 HIV-1 infected, ART naïve, asymptomatic adults and 20 HIV-1 uninfected adults were compared with respect to proportions and total number of leukocyte subsets in BAL obtained at bronchoscopy and in peripheral blood. The study is descriptive, providing a detailed profile of the leukocyte alterations associated with HIV-1 infection in these compartments.

 My comments are:

  

1. More details regarding whether there was appropriate matching of cases and controls should be provided. It is unclear whether co-morbidities and environmental exposures that may impact lung immune responses were well matched across arms. For example, were respiratory diseases or symptoms present in any participants? Was a history of environmental exposures taken (eg. indoor smoke, occupational)? Were participants asked about previous TB, current TB symptoms and was any TB diagnostic work-up undertaken in those with symptoms? Was there a chest X-ray done in participants? Was any testing for TB infection (“latent TB”) undertaken?
2. The term alveolitis is used in the title. “Alveolitis” suggests a clinical syndrome characterized by lung inflammation focused on the alveoli (as is the case with CD8+ alveolitis in HIV-infected patients – this manifests with a clinical syndrome). The authors have described increased numbers of certain immune cell types in the alveoli. I do not think this represents alveolitis and suggest changing this term in the title.
3. In the abstract it is stated that HIV “does not lead to massive depletion of alveolar CD4+ T cells”. This is true for the participants studied but most had CD4 count in blood > 200 thus this statement should be qualified by recognising this may not apply to patients with severe CD4 depletion in blood.
4. In the Methods, the delay between taking the BAL sample and processing should be described. This may affect cell viability, especially neutrophils.
5. On page 3 for this statement: “In PBMCs, the absolute numbers were obtained by calculating the proportion of a particular subset relative to the full blood count (FBC) data.” Suggest providing details about which cell populations from the FBC data were used for denominators in calculating cell numbers from the FACS data.
6. Page 7: “In blood, we found that the numbers of CD14+ CD16lo classical mono- cytes (median 110 vs. 60 cells/1000 mm3, p=0.0237), CD14+CD16+ intermediate monocyets (median 20 vs. 6 cells/1000 mm3, p=0.0362) and CD14loCD16+ non classical monocytes (median 10 vs. 30 cells/1000 mm3, p=0.0316) were higher in HIV-infected adults compared to HIV-uninfected controls.”  … It appears the non classical monocytes were lower (10 vs 30) in HIV-infected adults not higher. Please check this.
7. Page 10: “We have shown preserved mucosal CD4+ T cells in BAL fluid from chronic HIV-infected adults, even in those with depleted peripheral blood CD4 + T cells.” The statement I underlined should be justified by data presented. Was a stratified analysis of those with CD4<200 or <350 conducted?
8. The authors acknowledge that the accuracy of measurement of cell numbers in BAL may be inaccurate because of variations in the dilution factor of epithelial lining fluid and differences in BAL fluid volume return. They do not provide details on any methods they used to standardise this measurement across participants. For example in the BAL sampling technique or in standardising to a constituent of ELF. Suggest discuss this in more detail.

 

**Additional comments from my colleague Dr Muki Shey:**

**General comments**

 I was not certain whether clinically the presence of immune cells in the lungs of (HIV-infected) individuals constitute alveolitis, otherwise definition and contextualization was missing.

 There is no mention of TB infection in the participants or location where participants were recruited. Was there any screening for TB?

 Some more discussion is necessary about the differences between cell numbers and proportions in BAL and Blood.

**Specific comments**

- Figure 1 needs revision: Different antibody fluorochromes are given in method section while different ones appear in the figure. For example, CD3 PE-Cy5 vs PerCP Cy5.5; and CD45 PE-Texas Red s CF594 (even though the two fluorochromes may be detected in the same channel, there needs to be consistency)
- Figure1: The CD4/CD8 axes labelling seems to be reversed. I am quite certain that the frequencies of CD4 ^+^ T cells cannot be more than frequencies of CD8 ^+^ T cells in HIV infected individuals
- The TCR Va chain is mentioned in the methods section while the TCR gd appears in the plots.
- The gating of CD45 ^+^ cells looks more stringent in Figure 1 (for T cells) than in Figure 4 (for myeloid cells). Was there any reason for that?
- The definition of different cell subsets by expression of markers may be necessary, for example the definition of NK T cells (CD3 ^+^CD56 ^+^?)
- In the results, giving the various median values in the text which are in the figures seems a duplication.
- HLA-DR antibody is mentioned in methods section but not shown either as a marker of activation or identification of myeloid cells. HLA-DR is an important marker of myeloid cell identification, especially in whole blood.

We have read this submission. We believe that we have an appropriate level of expertise to confirm that it is of an acceptable scientific standard, however we have significant reservations, as outlined above.

# Response

**Published:** 2017-12-15

**Authors:** Kondwani Jambo

**Affiliations:** Malawi-Liverpool-Wellcome Trust Clinical research programme, Malawi

Thank you for the helpful comments. We have revised the manuscript to incorporate your suggestions.

Specifically, we have revised the title of manuscript, provided more details in the methodology section and corrected errors in the results section.

# Peer review: Referee response for version 1

**DOI:** 10.21956/wellcomeopenres.13949.r27370

**Published:** 2017-10-30

**Authors:** David H. Dockrell

**Affiliations:** 1 MRC Centre for Inflammation Research, University of Edinburgh, Edinburgh, UK

Mwale and co-workers examine the cellular composition of BAL asymptomatic HIV-positive Malawians. They show increased numbers of CD8 T-cells, B-cells and gd T-cells, while also identifying a reduction in classical monocytes in HIV which resulted in an increased proportion but not absolute number of intermediate monocytes. The data is important and adds a significant contribution to the literature, in particular through providing clinically relevant samples in HIV in patients naïve to ART. While the data makes a valuable contribution to the literature some areas of further detail would be informative.

  

1. Background demographic data shows the control group are well matched in terms of age and gender. More details on the HIV-positive group are required. What steps were taken to exclude TB. Is there any data on baseline CXR or TB screening or is the assumption that patients were negative purely based on lack of symptoms? Is there any data on baseline HIV viral load?
2. Some further methodological details would aid interpretation. While the differences in proportions of cell subsets are clear interpretation of absolute numbers requires evidence that volumes of instillation and BAL recovery are similar. Can the authors report their standard methodology involved instillation of 200 mls in four aliquots, into the right middle lobe, or whatever? Have they specific data on the volume recovered ad its variability. Can they confirm there were low (<5%) numbers of bronchial epithelial cells or squamous cells?
  Although the focus is analysis of cellular components have they any information on the permeability of the alveolar space in the two groups through measurement of albumin or a related marker? The authors appropriately remark in the discussion that these considerations limit interpretation of absolute numbers so this comment is meant only to provide detail not as a significant criticism.
3. The absolute number of monocytes in BAL should also be reported. In the abstract, the primary finding is presented as an increase in the proportion of intermediate monocytes in HIV BAL but in reality, the main finding appears to be a reduction in absolute number of classical monocytes which results in a relative rather than absolute increase in intermediate monocytes in HIV. Subsets of intermediate monocytes may also be defined by HLA-DR and the authors appear to have also used antibodies against HLA-DR. Did they find any differences in intermediate subsets by HLA-DR in their HIV positive population?
4. While reductions in non-classical monocytes are described by many groups the magnitude of the reduction in numbers of non-classical monocytes in the BAL is a little surprising since these cells are thought to be a source of alveolar macrophages. The authors suggest they can detect very few classical cells in BAL in contrast to the blood. Some more discussion of this point seems needed. Have the authors any data with alternative markers e.g. CCR2, CX3CR1 to confirm such low numbers? Or may they be missing some non-classical monocytes? This finding should be developed further and discussed a little further and related to other BAL-specific lung data.
5. In the discussion the authors highlight the different origins of monocytes and lung macrophages but some qualification of the differences in origin of lung macrophages in inflammatory settings and the
  potential for classical monocytes to contribute to lung macrophage numbers in inflammation may be pertinent.
6. In Figure 5D the HIV + dot has been labelled HIV – and needs to be altered.

I have read this submission. I believe that I have an appropriate level of expertise to confirm that it is of an acceptable scientific standard, however I have significant reservations, as outlined above.

# Response

**Published:** 2017-12-15

**Authors:** Kondwani Jambo

**Affiliations:** Malawi-Liverpool-Wellcome Trust Clinical research programme, Malawi

Thank you for the helpful comments. We have revised the manuscript to incorporate your suggestions.

 Specifically, provided more details in the methodology section and added more information in the discussion section.
